# Parturient perineal distensibility tolerance assessed by EPI-NO: an observational study

**DOI:** 10.1590/S1679-45082014AO2944

**Published:** 2014

**Authors:** Mary Uchiyama Nakamura, Nelson Sass, Julio Elito, Carla Dellabarba Petricelli, Sandra Maria Alexandre, Edward Araujo, Miriam Raquel Diniz Zanetti

**Affiliations:** 1 Escola Paulista de Medicina, Universidade Federal de São Paulo, São Paulo, SP, Brazil

**Keywords:** Pelvic floor/instrumentation, Perineum, Pregnant women, Parturition

## Abstract

**Objective:**

To determine how parturient women tolerate the use of a perineal distensibility assessment technique using the EPI-NO device.

**Methods:**

An observational study with a total of 227 full-term parturient women was performed. During the evaluation with EPI-NO, parturient patients were asked about their sensation of discomfort. The degree of discomfort was measured using the Visual Analogue Scale, with a score from zero to 10. The Mann-Whitney test was applied to assess perineal distensibility measured by EPI-NO and the degree of discomfort caused by the test according to parity. The relation between perineal distensibility and discomfort was analyzed by using the Spearman correlation test (r).

**Results:**

The test with EPI-NO caused only slight discomfort (mean Visual Analogue Scale of 3.8), and primiparous women reported significantly greater discomfort (mean Visual Analogue Scale of 4.5) than did multiparous (mean Visual Analogue Scale=3.1), with p<0.001 women. A negative correlation was observed, in other words, the greater the perineal distensibility on the EPI-NO, the lower the pain reported by the patients (r=-0.424; p<0.001).

**Conclusion:**

The assessment of perineal distensibility with EPI-NO was well tolerated by the parturient women.

## RESUMO

**Objetivo:**

Determinar como a mulher parturiente tolera o uso de uma nova técnica de extensibilidade perineal, por meio do aparelho EPI-NO.

**Métodos:**

Estudo observacional com um total de 227 gestantes a termo. Durante a avaliação pelo EPI-NO, as parturientes foram perguntadas sobre a sensação de desconforto. O grau de desconforto foi medido usando a Escala Visual Analógica, com escore entre zero a 10. O teste de Mann-Whitney foi usado para avaliar a extensibilidade perineal avaliada pelo EPI-NO e o grau de desconforto causado pelo teste de acordo com a paridade. A relação entre extensibilidade perineal e desconforto foi avaliada pelo teste de correlação de Spearman (r).

**Resultados:**

O teste com EPI-NO causou apenas leve desconforto (média da Escala Visual Analógica de 3,8), sendo que as primíparas reportaram mais desconforto de modo significativo (média da Escala Visual Analógica de 4,5) que as multíparas (média da Escala Visual Analógica de 3,1), com p<0,001. Observou-se correlação negativa, ou seja, a maior extensibilidade no EPI-NO foi acompanhada de menor dor referida pelas pacientes (r=-0,424; p<0,001).

**Conclusão:**

A avaliação da extensibilidade perineal com EPI-NO foi bem tolerada pelas parturientes.

## INTRODUCTION

The pelvic floor is currently the focus of several scientific studies, which largely concentrate on vaginal birth. Poor strength of the pelvic floor has been considered a predictive factor for complications after childbirth, such as urinary/fecal incontinence, and prolapsed genitals.^(1)^ Therefore, the stages of childbirth warrant further investigation as to their effects on the pelvic floor, especially regarding lesions such as lacerations and episiotomies.

An episiotomy is an indicated procedure in cases of fetal suffering, insufficient progression of labor, or when severe laceration is imminent. However, since the 1960s, doubts have been raised over the compulsory nature of the episiotomy. These questions stem from a lack of evidence of the benefits of episiotomies.^(2)^ According to the World Health Organization,^(3)^ episiotomies are classified as category D, that is, common practices used inappropriately.

The above information raises a question in clinical practice: how can parturient women who are likely to suffer severe laceration be identified? Lacerations occur when the soft or enveloping tissues, muscle, fascia, subcutaneous tissue, skin, and mucosa are not sufficiently elastic to allow fetal passage.^(4)^

In the absence of an instrument to objectively and quantitatively assess the degree of perineal distensibility, we adapted the EPI-NO as a method of measuring distensibility.

This is the first study using the EPI-NO inflatable balloon to measure perineal distensibility. The EPI-NO is introduced into the vagina and inflated to produce a distension of the pelvic floor muscles. An objective measure of muscular distensibility can be obtained by measuring the circumference of the fully inflated balloon. Although the device was not designed for this purpose, this adaptation was necessary because no alternative method of perineal distensibility assessment was available for use in obstetrics. This measurement could indicate perineal elasticity or stiffness and predict perineal integrity during labor.

## OBJECTIVE

To determine how a parturient tolerate the use of the perineal distensibility assessment technique with EPI-NO.

## METHODS

A cross-sectional observational study with a consecutive sample of parturient women was conducted between September and December of 2009 at the *Hospital Municipal e Maternidade Amador Aguiar* (HMMAA), at Osasco (SP, Brazil). This tertiary level hospital established a Natural Birth Centre in 2008 and records an average of 430 deliveries per month. This study was analyzed and approved by the Research Ethics Committee of the *Universidade Federal de São Paulo* (UNIFESP) under registration number 1283/08, and by the National Research Ethics Committee under report number. 676. The consent form was obtained from all patients included in this study.

We included 227 consecutive full-term parturient women carrying a single fetus in a vertex presentation and exhibiting up to 9cm of dilatation, according to the De Lee classification, at a maximum station of zero. Only collaborative parturient patients who wished to undergo the test and had not received analgesia (spinal, epidural, or combined - CSE), and whose fetus showed good vitality at the time of the assessment were included. Both primiparous and multiparous women were included in the study.

On admission to the delivery room, the participants underwent a perineal distensibility assessment, which measured the circumference (in centimetres) of the inflated EPI-NO balloon. This device constitutes a type of vaginal dilator consisting of an inflatable balloon connected to a manometer by a rubber tube. The silicone balloon has a figure-eight shape and its distal end is inserted into the vagina and then filled with air using the manometer.

All assessments were performed by the same examiner (MRDZ). The test was performed in parturient patients placed in prone position with lower limbs flexed and abducted (from 30° to 45°), and feet supported on the bed. The subjects were asked to not contract the gluteal, perineal, nor adductor muscles. After the application of a gel lubricant, the balloon, covered in a condom, was introduced into the vagina until 2cm were visible outside the vaginal introitus. The balloon was gradually inflated with the assistance of another professional until the tolerable limit determined by the patient was reached. At this point, the balloon was slowly withdrawn while still fully inflated, the condom was removed and the broadest circumference of the balloon was measured using a metric measuring tape. Immediately after completing the assessment, the parturient women were questioned about the degree of discomfort caused by the test. The degree of discomfort was measured using the Visual Analogue Scale (VAS) with a score from zero to 10, in which zero corresponded to no discomfort and 10 corresponded to maximal discomfort.^(5)^

Sample size was estimated in order to provide sufficient precision (95% confidence intervals – CI95% – width of 0.20)^(6)^ if the observed area under the receiver operating characters (ROC) curve was more than 0.60. Considering the area under the ROC curve as 0.713, we would need to assess 160 subjects to have a 95%CI width ≤0.20.

The data were analyzed using Excel 2007 (Microsoft Corp., Redmond, WA, USA) and the results are shown in the form of graphs and tables. A statistical description of the data was performed to demographically characterize the sample. The Mann-Whitney test was applied to assess perineal distensibility (measured using EPI-NO) according to parity to analyze the degree of discomfort caused by the test according to parity. The relation between perineal distensibility (using EPI-NO) and discomfort caused by performing the test (according to the VAS, outlined above) was analyzed using the Spearman correlation test (r). Significance was set at p<0.05.

## RESULTS

A total of 227 parturient women were included in this study, all of whom underwent the EPI-NO test to assess their perineal distensibility during the dilatation period of childbirth. The mean maternal age was 24.1±5.5 years (range: 15 to 40). The mean of gestations was 2.0±1.6 (range: 1 to 11).

**Figure 1:**
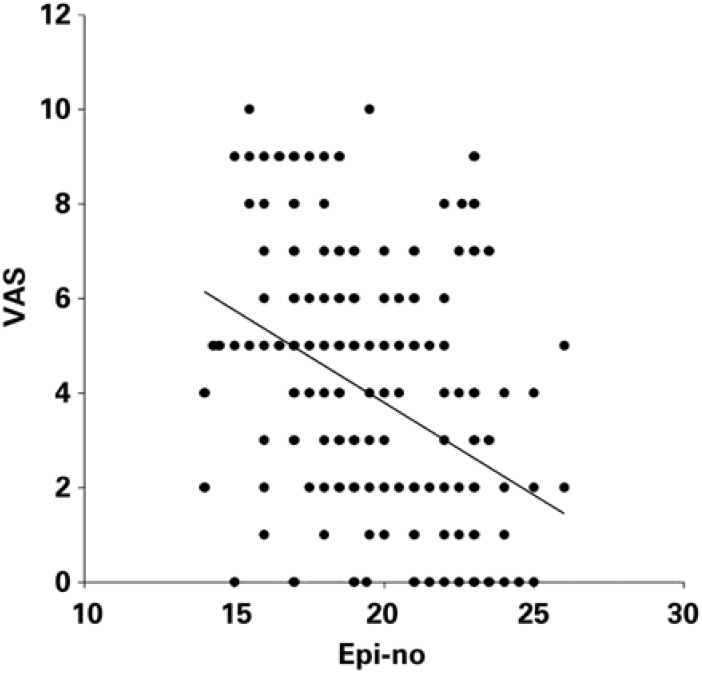
Scatter plot of correlation between Visual Analogue Scale and EPI-NO

A total of 48.5% (n=110) of the study subjects were multiparous, and 51.5% (n=117) were primiparous. The peripartum variables of the parturient patients are shown on table 1. The perineal distensibility test using the EPI-NO produced an average value of 3.8 on the VAS (Table 2). The Mann-Whitney test was performed to assess perineal distensibility according to parity and to analyze the degree of discomfort caused by the test according to parity (Table 2). Figure 1 shows an inverse relationship between perineal distensibility and the degree of discomfort reported by the parturient, based on the Spearman correlation test. A negative correlation was found (r=-0.424); the greater the perineal distensibility on the EPI-NO, the lower the pain reported by the patient (p<0.001).

**Table 1:** Description of peripartum variables

Variable	n	Minimun	Maximun	Mean	Standard deviation	Median
Circumference reached on EPI-NO (cm)	227	14.0	26.0	19.9	2.7	20.0
Visual Analogue Scale	227	0.0	10.0	3.8	2.6	4.0

**Table 2:** Comparison of perineal distensibility and degree of discomfort reported by primiparous and multiparous

Variable	Parity	n	Mean	Standard deviation	Minimun	Maximun	Median	p-value
EPI-NO (cm)	Primiparous	117	19.3	2.8	14.0	26.0	19.0	<0.001*
	Multiparous	110	20.7	2.5	14.0	26.0	21.0	
	Total	227	19.9	2.7	14.0	26.0	20.0	
Visual Analogue Scale	Primiparous	117	4.5	2.7	0.0	10.0	5.0	<0.001*
	Multiparous	110	3.1	2.4	0.0	10.0	2.0	
	Total	227	3.8	2.6	0.0	10.0	4.0	

* Mann-Whitney test.

## DISCUSSION

The maximum length of most striated muscles can be indirectly assessed by measuring the angle of amplitude of the maximum passive movement of a given joint. The amplitude of movement is measured with the aid of a goniometer and depends on joint mobility and the mobility and flexibility of the joint-related tissues (muscles, connective tissue, and skin).^(7)^

Measurement of the maximum length of pelvic floor muscles is extremely limited because tension in these muscles does not directly affect the movement of any joint. Thus, a goniometer is not useful in assessing whether the musculature of the pelvic floor is stiff or not particularly extensible. Although the EPI-NO device was not designed to measure perineal distensibility, this adaptation was necessary because no alternative method was available.

The 227 parturient women in this study had a mean age of 24.1 years, and most were experiencing their first or second pregnancy. Parity analysis showed the sample to be equally split between multiparous and primiparous patients. The mean perineal distensibility assessed in the 227 subjects was 19.9cm, based on the balloon circumference of the EPI-NO. Because this study was the first study to assess perineal distensibility without previous intervention, there are no published parameters with which to compare our measurements. However, considering that the full-term fetal head circumference ranges between 33 and 35cm, we believe our mean perineal distensibility measurement to be low.

Although the head circumference of newborns is well established, the fetal cranial bones move and overlap, what is facilitated by the fontanells, at the time of detachment of the cephalic pole from the pelvis during delivery. Therefore, head circumference at this stage may be significantly reduced.

Recently, Ruckhäberle et al.^(8)^ conducted a prospective, randomized study using EPI-NO during gestation for perineal preparation (increase in muscular distensibility) prior to childbirth. The study group consisted of 135 primigravids who used the device for at least 15 minutes per day from the 37th gestational week (for an average of 15 consecutive days), and the control group consisted of 135 primigravids with no perineal preparation. The study group achieved a mean balloon circumference of 24.3±4.4cm after training and showed a significantly higher frequency (p=0.05) of intact perinea compared to the control group. The control group did not use any perineal preparation (neither EPI-NO), and the authors had no way of knowing the similarity in perineal distensibility between the groups before treatment.

In the present study, the balloon circumference attained by primiparous women was lower (19.3±2.8cm) than that of multiparous (20.7±0.5cm) women (p<0.001). The data from the multiparous group were lower than those reported by Ruckhäberle et al.^(8)^ However, in their study, the measurement was taken and reported by the pregnant patient, which could lead to a bias in results. Moreover, during labor the parturient patients are exposed to stress, which may lead to lower tolerance of EPI-NO balloon; this fact can justify the lower balloon circumference obtained in our study during labor.

Because this is a new method of measurement, it was necessary to check the level of tolerance by the parturient women of undergoing an additional intravaginal test and the degree of discomfort reported following the EPI-NO assessment. During the course of the study, we observed that a few parturient^(5)^ women with emotional decompensation often rejected even an obstetrician's digital evaluation; therefore, they were not invited to participate in the study.

Overall, the EPI-NO test caused only minor discomfort (VAS=3.8), considering that the scale varied from zero to 10. Because subjects presented different degrees of perineal distensibility, we compared the VAS results of multiparous with those of primiparous patients, which revealed that the multiparous group reported significantly lower discomfort (p<0.001). In addition, we noted that the greater the perineal distensibility, the lower the discomfort caused by the test (p<0.001).

Although we have found no test for the pelvic floor similar to the EPI-NO test, in assessments of other skeletal muscles, Magnusson et al.^(4)^ noted that the poorer the result on the Schober test, which indirectly assesses the distensibility of the ischiotibial muscles (*i.e.*, the greatest distance of the fingers from the ground during trunk flexion), the lower the stretch tolerance of the subject. So, apparently, all skeletal muscles, including pelvic floor muscles, present same behaviour concerning distensibility tolerance.

Episiotomy should be used to prevent severe lacerations, which are classified as third- and fourth-degree injuries to the external sphincter of the anus and rectal mucosa that cause much greater pain than minor lesions^(9)^, and are more strongly associated with faecal incontinence.^(10)^ Although right mediolateral episiotomy may play a protective role in severe lacerations, its liberal use should be discouraged, as it leads to complications such as significant bleeding, dyspareunia, pudendal nerve damage, and greater risk of major perineal lesions in a posterior delivery.^(11)^ Therefore, it is important to determine predictive factors of perineal integrity to establish when an episiotomy should not be used.

The ability of the pelvic floor muscles to distend varies among different parturient women and among different pregnancies in the same individual. This ability can be reduced or increased during the course of a pregnancy, promoting the shrinkage or stretching of these muscles, by using techniques such as perineal massage,^(12,13)^ which is well accepted.^(14)^

Pelvic floor muscular distensibility should be exhaustively studied to prevent major lesions during childbirth. This study provided evidence that the EPI-NO distensibility measurement method is well tolerated by patients; therefore, it could be used in obstetrical clinical practice.

The primary goal of this study was to investigate the tolerance of a new objective and quantitative approach for assessing perineal distensibility in parturient women using the EPI-NO device. The tolerance of its use was directly correlated to the patient's perineal distensibility, which is greater in multiparous than in primiparous patients.

## CONCLUSION

Based on the results of this study, we conclude that the assessment of perineal distensibility using the EPI-NO device is well tolerated by parturient women and could be used at the time of parturition. The EPI-NO device highlights perineal distensibility during labor, thus reducing the risk of lacerations and the need for an episiotomy.
